# Using data to support evidence-informed decisions about skilled birth attendants in fragile contexts: a situational analysis from Democratic Republic of the Congo

**DOI:** 10.1186/s12960-020-00511-w

**Published:** 2020-10-29

**Authors:** Amuda Baba, Tim Martineau, Sally Theobald, Paluku Sabuni, Joanna Raven

**Affiliations:** 1 Institut Panafricain de Santé Communautaire, Aru, Democratic Republic of the Congo; 2 Liverpool School of Tropical Medicine, Liverpool, UK; 3 Université Officielle de Rwenzori, Butembo DR Congo and the Leprosy Mission, Kinshasa, Democratic Republic of the Congo

**Keywords:** Skilled birth attendants, Nurses, Midwives, Data, Fragile contexts, DRC

## Abstract

**Background:**

Most low- and middle-income countries are experiencing challenges in maternal health in relation to accessing skilled birth attendants (SBA). The first step in addressing this problem is understanding the current situation. We aimed to understand SBA’s availability and distribution in Ituri Province, North Eastern Democratic Republic of the Congo (DRC) from 2013 to 2017.

**Methods:**

We used available data on SBAs (doctors, nurses and midwives) from the Ituri Provincial Human Resource for Health Management Unit’s database from 2013 to 2017. The current distribution across and within three categories of district (rural, peri-urban and urban) and characteristics of SBAs as well as 5-year trends and vacancy trends were identified. Data on training outputs for SBA cadres was collected from training schools in the province. Descriptive analysis, disaggregating by district, cadre and gender where possible, was conducted using Excel.

**Results:**

The national ratio of SBAs per 1000 population is four times less than the Sustainable Development Goals threshold (4.45) while the Ituri Province ratio is one of the lowest in DRC. There are more doctors and nurses in urban and peri-urban districts compared to posts, and shortages of midwives in all district categories, particularly in rural districts. From 2013 to 2017, occupied posts for doctors and nurses in all three categories of districts increase while midwives decrease in peri-urban and rural districts. There is clear gender and occupational segregation: doctors and nurses are more likely to be male, whereas midwives are more likely to be female. The projections of training outputs show a surplus against authorised posts of doctors and nursing increasing, while the shortfall for midwives remains above 75%.

**Conclusion:**

This is the first study to use existing human resource data to analyse availability and distribution of SBAs in a DRC province. This has provided insight into the mismatch of supply and demand of SBAs, highlighting the extreme shortage of midwives throughout the province. Further investigations are needed to better understand the situation and develop strategies to ensure a more equitable distribution of SBAs throughout this province and beyond. Without this, DRC will continue to struggle to reduce maternal mortality.

## Introduction

Around 300 000 women die from preventable causes related to pregnancy and childbirth every year [1]. Low- and middle-income countries (LMICs) were responsible for 99% of global maternal deaths, and sub-Saharan Africa accounted for 66% of those deaths [2, 3]. There is a global health workforce crisis with sub-Saharan Africa being severely affected by both availability and distribution of health workers [4, 5]. It is recognised worldwide that skilled birth attendance is one of the most effective strategies to reduce maternal mortality in LMICs, yet there are challenges with accessing qualified health workers and services [6–8]. A skilled birth attendant (SBA) is defined by WHO as “an accredited health professional — such as a midwife, doctor or nurse — who has been educated and trained to proficiency in the skills needed to manage normal (uncomplicated) pregnancies, childbirth and the immediate postnatal period, and in the identification, management and referral of complications in women and newborns” [9]. Doctors, nurses and midwives are the cadres recognised as skilled birth attendants in most countries, including the Democratic Republic of the Congo (DRC) [10–12].

Availability of SBAs appears to be particularly seriously affected in fragile and conflict-affected settings (FCAS), which are responsible for one third of maternal deaths worldwide [13, 14]. This places a much higher burden on the few SBAs in already demanding environments and limits the availability of quality health care services [14].

DRC is characterised by incessant political turbulence from its Post-Independence period in the 1960s [15, 16]. In the early 1990s, DRC again experienced decades of political instability and conflicts, affecting all sectors, including health [17, 18]. With a maternal mortality rate of 846 per 100,000 live births as well as facing a series of crises, DRC is classified by the Department for International Development [18] as being in a situation of “high fragility” [19, 20]. Ituri Province (the study setting) is mostly rural and is going through sustained socio-political crises and wars since 1999. Ethnic clashes and the recent Ebola outbreak make this province particularly fragile [21, 22]. Ituri Province faces challenges in attracting and retaining midwives in rural districts and has a high maternal mortality rate far beyond the national average as in some rural provinces in DR Congo [23–25].

Skilled birth attendants’ training in DRC is managed by two different ministries: those following the secondary level of nursing school (diploma) (A2), i.e. 4 years after 10 years of education, are managed by the Ministry of Public Health, whereas those studying nursing or midwifery degrees at nursing colleges (A1: undergraduate degree or A0: post graduate degree) and medical doctors at faculty of medicine at universities are under the management of the Ministry of Higher Education (see Table 1) [11, 26, 28]. Doctors, nurses and midwives spend respectively 25%, 40% and 95% of their time on maternal and newborn health [10].

**Table 1:** Training of different SBA cadres in DRC by government ministry

Ministry of Public Health (MoPH)	Ministry of Higher Education (MoHEd)
1. Nursing training *A2 nurses (Diploma)* - 10 years of education (6 years of primary and 4 years in secondary) - 4 years of nursing in nursing schools	1. Nursing training*.* *A1 nurses (undergraduate degree in nursing)* - A2 nurses or 12 years of education (6 years in primary and 6 years in secondary schools) - 3 years of nursing in nursing colleges *A0 Nurses (Post graduate degree in nursing)* - A1 nurses - 2 years of training in nursing for graduate degree
2. Midwifery training *A2 midwives (diploma level)* - 10 years of education (6 years of primary and 4 years in secondary) - 4 years of midwifery in nursing schools	2. Midwifery training *A1 midwives (undergraduate degree)* - A2 midwives or 12 years of education (6 years in primary and 6 years in secondary schools) - 3 years of midwifery in nursing colleges *A0 midwives (Post graduate degree)* - A1 midwives - 2 years of training in midwifery for post graduate degree 3. Physicians training - 12 years of education (6 years of primary education and 6 years of secondary schools) - 8 years of medical studies in the faculty of medicine

Adapted from Bansele and Hatem et al. ([26, 27])

Despite the fact that DRC produces an average of 2000 doctors and 7000 nurses yearly [29], it is known that there is a general severe shortage of doctors, nurses and midwives, with only 1.05 doctors, nurses and midwives per 1000 population, far below the Sustainable Development Goals indicative threshold of 4.45 doctors, nurses and midwives per 1000 population [30]. While the absolute shortages of SBAs are clear, little is known how many SBAs there should be, i.e. number of posts versus posts filled and how they should be distributed between urban and rural areas. We started our investigation using available routine data. This evidence, combined with data on local training output, will help to support evidence-informed decisions about doctors, nurses and midwives in fragile contexts and also inform strategies to develop the workforce to address maternal mortality challenges in Ituri Province.

## Methods

### Study design

**Fig. 1:**
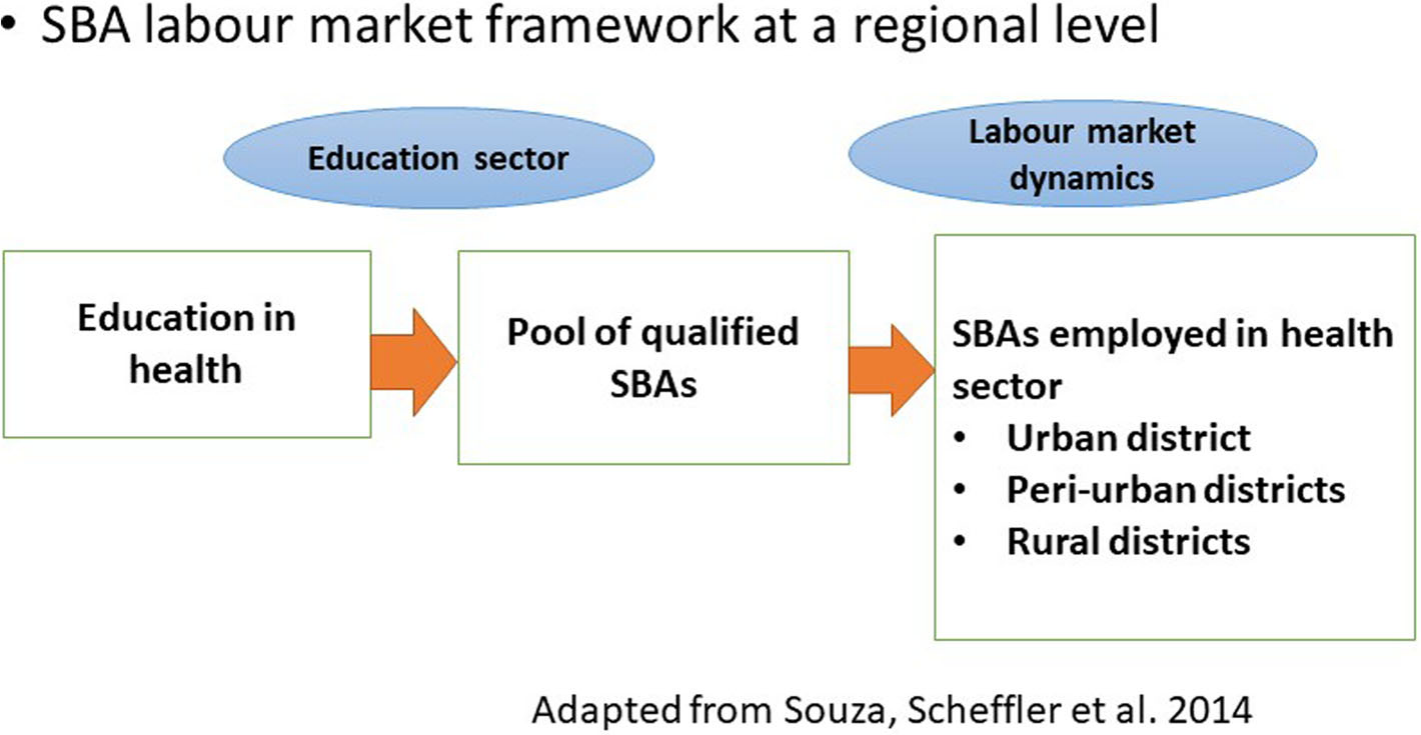
SBA labour market framework at a regional level

This study was conducted to understand how data can be used to support evidence-informed decisions about doctors, nurses and midwives in fragile contexts in Ituri Province, in North-Eastern DRC. This evidence, or the methods of deriving it using available data, may also be useful for other parts of DRC and other countries. The situation analysis critically reviewed secondary data from Ituri Provincial Health Division. An adaptation of the health labour market framework, as described by Sousa et al. [31] , was applied to SBA flows in the 36 districts in Ituri (see Fig. 1). The areas covered: health training and the entry of graduates into a pool of qualified SBAs as part of the “education sector”, and the distribution of graduates employed in the health sector in Ituri by type of district as part of the health sector labour market. Attrition neither from training into employment in the health sector nor attrition from the health sector could be covered, due to lack of data.

### Study setting: Ituri Province

**Fig. 2:**
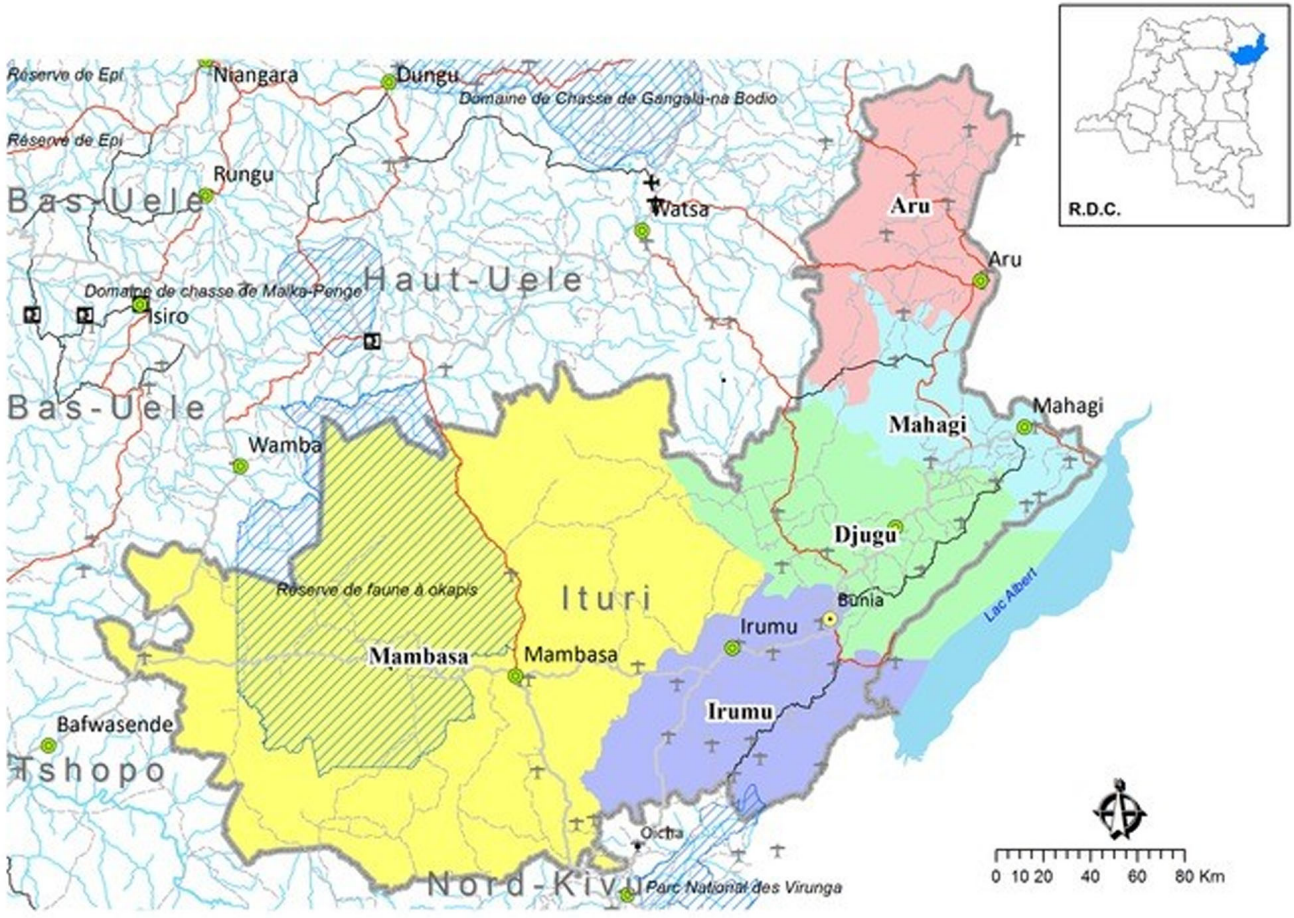
Map of Ituri Province. Source: Cellule d’Analyse des Indicateurs de Développement [32]

Ituri is one of the 26 provinces of DRC, covering an area of 65 293 km^2^, and has 36 health districts (see Fig. 2). We used the Provincial Health Division’s categorisation of districts (Table 2) [25]: the urban district, where the Provincial capital is found (1); the peri-urban districts, those located where there are concentrations of people, with some facilities in remote areas (7); and rural districts, those having all facilities in rural and remote areas (28).

**Table 2:** Categories of health districts in Ituri Province

Urban district	Peri-urban districts	Rural districts		
1. Bunia	1. Ariwara 2. Aru 3. Mahagi 4. Mambasa 5. Mandima 6. Mongbwalu 7. Niania	1. Adi 2. Adja 3. Angumu 4. Aungba 5. Bambu 6. Biringi 7. Boga 8. Damas 9. Drodro 10. Fataki	11. Gety 12. Jiba 13. Kambala 14. Kilo 15. Komanda 16. Laybo 17. Linga 18. Lita 19. Logo 20. Lolwa	21. Mangala 22. Nizi 23. Rethy 24. Tchomia 25. Nyankunde 26. Nyarambe 27. Rwampara 28. Rimba

Source: DPS Ituri [25], adapted by authors

### Data collection

District health officers are required to submit staffing returns to the Provincial Health Office every 6 months which is then entered into the HRH database. Using a pre-designed tool, we collected data on the number of established posts for doctors, nurses and midwives, and the number of posts filled by cadre and gender from the HRH database for 2015 to 2017 only. As the Provincial Health Office was established in Bunia, the capital of the Province, in early 2015 and the staffing database did not exist prior to this period, there was no data for years 2013 and 2014. Therefore the Provincial Human Resource for Health Management Unit analyst contacted the District Health Officers for this data which they inserted into the tool. Data does not include staffing from private health facilities.

Data on SBA graduates from 17 nursing schools were also collected at the Provincial Health Office to establish the training output. Data for degree graduates were collected from three nursing colleges and the Faculty of Medicine of Bunia University in the Province, to understand the trend of nurses, midwives and doctors trained each year from 2013 to 2018.

Data on SBA cadres joining and leaving each year were not available at the Provincial HRH Management Unit nor the health district level.

### Quality assurance

All the data related to different skilled birth attendants were received from Provincial HRH unit and from different schools producing SBAs. Once received, data from each health district, nursing school, nursing college and the faculty of medicine of the University of Bunia were systematically verified by checking the hard copy against the electronic copy in their database. The data was then inserted into the Excel file to prepare for analysis. All data were kept in both a password-secured computer and a password-secured external drive owned by and only accessed by the first author. The results were shared with provincial and the 3 district health authorities for their comments.

### Analysis

The collected data were compiled in a predesigned tool and a descriptive analysis, disaggregating by health district categories, cadres and gender where available, was conducted using Excel. For different SBA cadres, we calculated the percentage of posts filled as the total number of filled posts multiplying by 100, and dividing that result by the total number of established positions. Then, trends for different SBA cadres were analysed from 2013 to 2017. In relation to training outputs, the trends of different SBA cadres produced from nursing schools, colleges and the faculty of medicine were also analysed from 2013 to 2018 in order to make possible projections, and matching the training outputs to the shortages for different cadres. A special focus was also given to matching training outputs to shortages of SBAs.

### Ethics

Ethical approval was received from the Liverpool School of Tropical Medicine (Research protocol 17-024) and the Multidisciplinary Research Centre for Development in Bunia, DRC (018/2017). No names or identifying characteristics were collected.

## Results

This section starts by placing the data on SBAs in the global and national contexts. It then examines staffing in Ituri Province comparing the authorised posts with filled posts. The data is explored by cadre and type of district, and by gender where possible, especially in relation to posts filled in the districts. Finally, we examine the impact that training output at current rates could have on availability of SBAs.

### Global and national picture

#### SBA cadres per 1000 population: fewer than required

DRC has a ratio of 1.05 doctors, nurses and midwives per 1000 population. This is below the sub-Saharan African average of 1.2 per 1000 population, and far below the Sustainable Development Goals threshold of 4.45 doctors, nurses and midwives per 1000 population (Table 3). Neighbouring countries to DRC also experience a low density of health workers, for example, Angola and Republic of Congo have higher densities than DRC at 1.5 and 1.8, whereas Central Africa Republic, Burundi and Uganda have lower densities.

**Table 3:** Doctors, nurses and midwives per 1000 population in selected African countries

Countries	Staff to population ratio (doctors, nurses and midwives per 1000 population)
Republic of Congo	1.8 (2011)
Angola	1.5 (2017)
DRC	1.05 (2015)
Zambia	1.0 (2016)
Rwanda	0.9 (2017)
Burundi	0.8 (2016)
Uganda	0.7 (2015)
Central Africa republic	0.3 (2015)
Sub-Saharan Africa	1.2 (2015)
SDG Threshold	4.45 (2016)

Source: Banque Mondiale ([33, 34])

Ituri Province has a density of 0.526 health workers per 1000 population (Table 4), which is almost half DRC’s national average density of 1.05. It is far lower than some provinces such as Kongo Central at 1.98 and neighbouring North Kivu (0.85).

**Table 4:** Doctors, nurses and midwives per 1000 population in selected provinces in DRC

Province	Staff to population ratio (doctors, nurses and midwives per 1000 population)
Kongo Central	1.98 (2013)
Maniema	1.24 (2013)
Kinshasa	0.90 (2013)
North Kivu	0.85 (2013)
South Kivu	0.83 (2013)
Ituri	0.526 (2017)
Average in DRC	1.05 (2015)

Source: Division Provinciale de Santé Ituri, Ministère de la Sante Publique RD Congo ([11, 25])

### Distribution of SBAs within Ituri Province

We now investigate the data on SBAs in Ituri Province. We start by reporting on the number of staff in the different SBA cadres there should be by type of district from data collected for 2017 (see Table 5).

**Table 5:** Number of posts by cadre and type of districts in 2017

	Urban district		Peri urban districts		Rural districts		All districts	
Cadres	Authorised posts	Posts filled (% of posts filled)	Authorised posts	Posts filled	Authorised posts	Posts filled	Authorised posts	Posts filled
Doctors	18	43 (238.9%)	60	61 (101.7%)	150	137 (91.3%)	228	241 (105.7%)
Nurses	185	348 (188.1%)	508	632 (124.4%)	1776	1572 (88.5%)	2469	2552 (103.4%)
Midwives	75	69 (92.0%)	269	67 (24.9%)	1039	75 (7.2%)	1383	211 (15.3%)
Total	278	460	837	760	2965	1784	4080	3004

#### Distribution of SBAs in 2017: severe shortage of midwives

Breaking down the analysis of where the shortages lie shows an intriguing picture (see Table 5). The shortages of midwives are the most extreme, especially in peri-urban (24.9% of posts filled) and rural districts (7.2% of posts filled). This is the cadre whose training, and it can be assumed whose job roles, is almost exclusively focused on maternal and newborn health. In contrast, 88.5% of the posts of nurses in rural districts are filled. Since the number of posts filled for midwives across the 28 rural and 7 peri-urban health districts is comparatively low, respectively 75 in rural health districts (7.2%) and 67 in peri-urban health districts (24.9%), it can be assumed that if any maternal and newborn health services are being provided, this is being done by nurses.

A second point to note from this data is that although the ratio of SBAs to population is half that of the national average, there is an overall surplus of posts filled versus authorised posts for both doctors (105.7%) and nurses (103.4%). There are more nurses than the authorised posts in peri-urban districts (124.4%) and especially in Bunia, the urban district (188.8%). This corresponds to an 11.5 % shortage of nurses in rural districts. There are also more doctors in urban district (238.9%) compared to the authorised posts and a small shortage in the rural districts (8.7%). In theory, if the extra number of nurses and doctors in urban and peri-urban districts were redistributed to rural districts, the shortage against approved posts would be eliminated.

#### Distribution of SBAs over time (2013–2017): nurses and doctors are increasing, leaving midwives behind

**Fig. 3:**
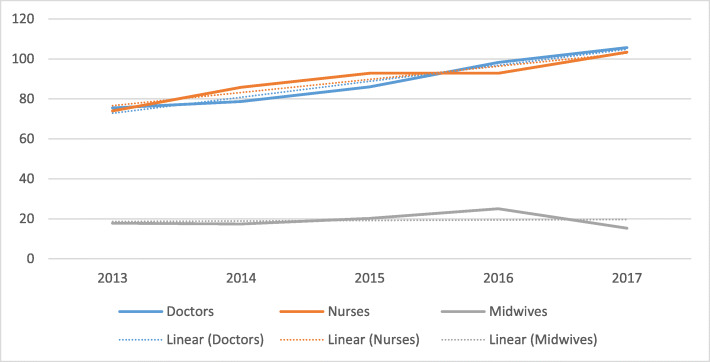
Percentage of SBA posts filled in Ituri Province from 2013 to 2017

In the previous section, we looked at a static position on distribution. It is important to examine the dynamic situation to establish whether the situation is getting better or worse. The following charts are derived from the collected data series (Fig. 3). The overall picture in Ituri Province is that all cadres are increasing, though only very minimally for midwives.

#### Distribution of SBAs across the categories of districts: filled posts are lowest in rural districts

**Fig. 4:**
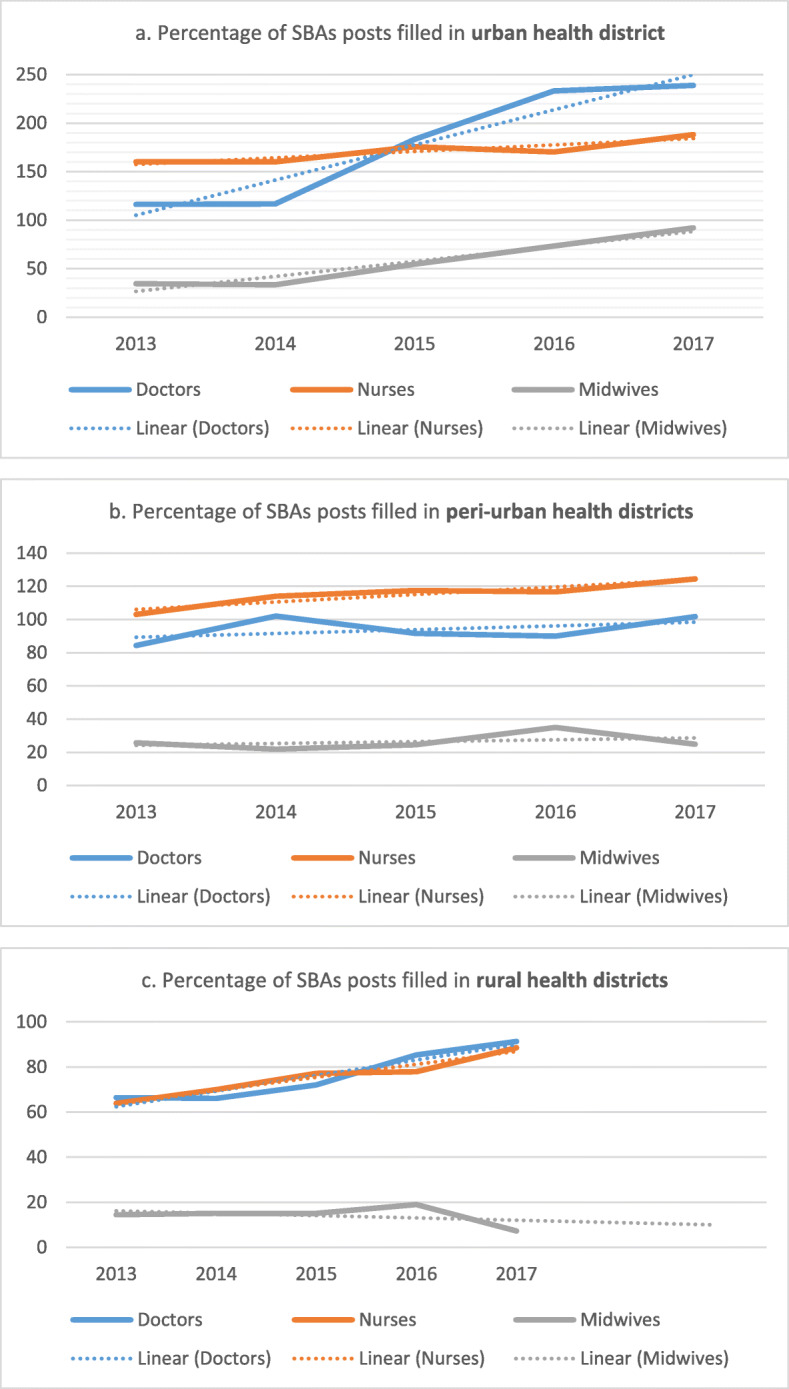
**a** Percentage of SBA posts filled in urban district from 2013 to 2017. **b** Percentage of SBA posts filled in peri-urban districts from 2013 to 2017. **c** Percentage of SBA posts filled in rural districts from 2013 to 2017

In Bunia, the only urban health district, the increase for midwives from 2014 to 2017 has been quite steep (from 25 to 69 making an increase of 176%)—see Fig. 4a. The increase for nurses has been more modest (17.6%), though there was already more nurses compared to the number of authorised posts (160%) in 2013. The number of authorised posts for doctors in Bunia has remained at 18 since 2013, but the actual number of filled posts has risen from 21 to 43 (104.8% increase) since then.

Filled posts for doctors and nurses rose slightly in the 7 peri-urban districts (see Fig. 4b) and in 28 rural districts (see Fig. 4c). The number of authorised posts for doctors also rose by 14.5% from 131 in 2013 to 150 in 2017, while the percentage of filled posts rose from 66.4 to 91.3% in the same period. The contrast, however, is with midwives. The serious shortfall reported above in these districts appears to be part of a slight downward trend from 69 posts filled in 2013 to 67 posts filled in 2017 in peri-urban health districts and from 147 posts filled in 2013 to 75 posts filled in rural health districts, though there was a peak of 94 in peri-urban and 197 in rural health districts in 2016.

#### Distribution of SBAs in 2017 by gender

##### More male doctors and nurses

**Fig. 5:**
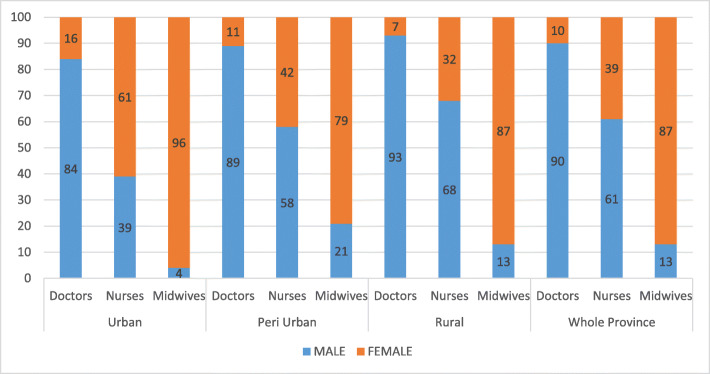
Ituri SBA cadres disaggregated by district categories and gender (percentage)

Another dimension of distribution of health workers is gender, with female health workers less likely to work in rural areas as described in some countries affected by conflict [35]. The routine data allowed for an analysis of the three cadres by gender and location (see Fig. 5). In all three categories of district, there are more male doctors and female midwives. The picture for nurses is more complicated. Despite 61% of nurses being male in the province (see Fig. 5), a situation different from other countries, where most nurses are female [36], less than 1/3 of nurses in rural health districts are female, while in the urban health district they make up 61% of nurses. As you move from urban to peri-urban and rural districts, the proportion of female nurses reduces.

### Potential supply of SBAs in Ituri

**Fig. 6:**
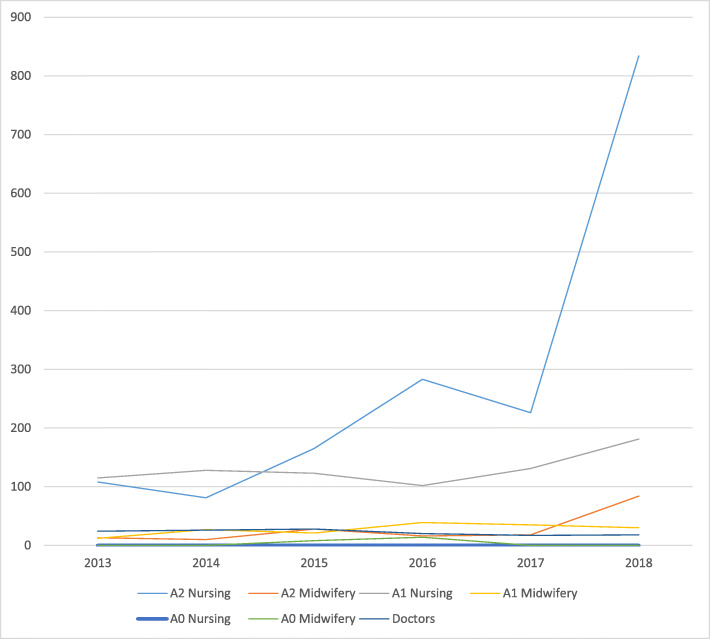
Graduates from nursing schools, colleges and the faculty of medicine 2013 to 2018

While the production of A2 nurses, A1 nurses and A2 midwives in Ituri Province shows an increase in 2018, that of A1 midwives, A0 nurses, A0 midwives and doctors remains almost static (see Fig. 6).

Working from the available data, which does not include how many graduates trained in Ituri enter service in the province, we can only make crude observations. Assuming no attrition from the pool of graduates to employment nor from the existing stock of health workers, we can estimate the impact of the training output from 2018. The number of doctors would increase by 17 increasing the surplus (versus authorised posts) to 107.5%, and the number of nurses would increase by 1015 increasing the surplus (versus authorised posts) to a massive 144.5%. In contrast, the additional 114 midwifery graduates would only reduce the shortfall of posts filled from 84.7 to 76.5%. It would require the same training output for another 3 years, assuming no attrition from training or the existing stock of midwives to fill the full number of authorised positions.

## Discussion

This situation analysis was based on data from Ituri Provincial Health Division in North-eastern DRC to understand how data can be used to support evidence-informed decisions about nurses and midwives in fragile contexts. This study reveals that the national ratio of doctors, nurses and midwives per 1000 population is 4 times less than the Sustainable Development Goals threshold (4.45) while the Ituri Province ratio is one of the lowest in DRC at half of the national and hence 8 times less than the SDG threshold. The shortages of midwives are the most extreme, especially in peri-urban and rural districts. While the number of doctors and nurses has increased in all three district categories from 2013 to 2017, the number of midwives has decreased in peri-urban and rural districts. There are more male doctors and nurses and more female midwives in the whole province, and this trend applies for doctors and midwives in all three district categories, but there are more female nurses in the urban district (61%). The supply of nurses (A2 and A1) and midwives (A2) is increasing, while the production of doctors, A1 midwives and A0 midwives remains static.

### The need for data to understand the situation

When trying to address staffing problems, you need data as this will inform appropriate strategy development. Sometimes, and especially in FCAS, good data is difficult to get [37], because of damage to ministries and health facility infrastructure and challenges in collecting this data. However, you have to start with what data is available. In this study, the HRH unit managers took initiative to get further years of data which supported our trend analysis. Without data on recruitment and retention, we were not able to fully understand the workforce dynamics. Further data is needed to better understand the HR situation in the province. We recommend some improvements on data collection, as suggested in the WHO minimum data set for health workforce registry, including estimation of workforce needs, pre-service and in-service training and information about exit, e.g. retirement, death, transfer, resignation and turnover [38]

### The mismatch in supply and demand of doctors, nurses and midwives

Even though the filled posts rates for doctors and nurses are beyond 100% at the provincial level, nursing schools and colleges, and the faculty of medicine continue to produce more nurses and doctors every year, overproduction of doctors and nurses is a challenge throughout DRC [11, 39, 40]. For instance, in 2013, 94% of students admitted to nursing schools in DRC followed nursing courses, and only 6% followed other courses, including midwifery, laboratory, pharmacy, hygiene and sanitation [11]. There are 406 nursing schools registered in DRC, yet only 16 provide midwifery courses [11] contributing to the shortage of this cadre. The number of graduating doctors has tripled from 2004 to 2015 (from 6000 to more than 18 000) [11]. The number of nursing schools and universities providing medical training has increased without regulation, which has implications for the quality of training [11, 40, 41]. This situation in the education sector (see Fig. 1) has contributed to the oversupply of nurses and doctors and the shortages of midwives in Ituri Province. Having established this situation from the routine data, it is now necessary to go beyond the numbers to get an in-depth understanding of why only few institutions provide midwifery courses and the reasons students select nursing rather than midwifery.

### Causes of shortage of midwives

The shortages of midwives are the most extreme, especially in peri-urban and rural districts. Further investigation is needed on the causes of shortages of midwives. A recent study on the lived experiences of midwives in Ituri Province showed that they face multiple challenges in their daily work including poor working conditions due to lack of equipment, supplies and professional support, which is made worse by the insecurity caused by militia operating in some rural districts [42]. In DRC, only a third of health workers receive salary from the government and half receive their risk allowances [11, 20]. In addition, salary is only a small proportion of the total remuneration of government health workers. The highest proportion comes from the share of user fees which are higher in urban areas [20]. Allowances that are targeted to health workers in rural facilities can help attract and retain health workers in rural areas by compensating for the lower earnings from user fees in rural areas [43]. Other studies have found that midwives in rural areas in sub-Saharan countries face challenges of long working hours as there are few staff in facilities, safety issues as staying overnight in facilities, poor access to in-service training and few opportunities for career progression [44]. Stress and burnout are common amongst rural midwives exacerbated by work overload due to understaffing, inadequate supply of drugs and supplies, insufficient protection from infections and poor remuneration [45]. These conditions are likely to lead to high levels of attrition, but may also deter school leavers to enrol in midwifery training and prefer training for nurses. The destination of the midwives who leave is unknown, but since the private sector is known to pay well and has better working conditions, they may be joining private-for-profit facilities. In addition, most private-for-profit facilities are located in urban areas or at the centre of peri-urban areas which midwives may find more desirable locations to serve.

### Gender norms and security concerns shape HR distribution

Across all 3 geographical contexts there is clear gender and occupational segregation: doctors and nurses are more likely to be male, whereas midwives are more likely to be female. There are slight differences between districts; in the rural and peri-urban areas, there are slightly more male nurses which probably reflects security concerns in these contexts, as described elsewhere [35]. These gendered patterns reflect those elsewhere—more male doctors, more female midwives [46, 47]. The exception in this context is the prevalence of male nurses. In the DRC, nursing arguably has greater career prospects—nurses can apply for a range of different jobs. Midwifery is also seen as a demanding and elastic role: midwives are expected to remain with women in labour until they deliver, which often means staying with them through the night. Men too can be put off applying for midwifery which is constructed as a feminised role (“Sage femme”, “Accoucheuse”), and male midwives may be rejected within rural communities as observed in some countries such as South Sudan, Mali, Afghanistan and Ghana [35]

### Deployment processes: more doctors and nurses in urban and peri-urban districts

The implication of having more nurses and doctors compared to the authorised posts in peri-urban and urban areas is that the deployment systems (initial posting and transfers) are not working properly. Some doctors and nurses appear to be able to select where they work, despite the need for posts to be filled in other areas. This is not unique to DRC, but is happening in many other settings within sub-Saharan Africa [43, 48]. The Provincial Health Office can review the human resource policies on deployment and the implementation of the policies to identify what actions could be taken to achieve more equitable staffing.

### Implications of shortages of midwives and maldistribution

The extreme shortage of midwives shown in this study, particularly in the rural areas, suggests that other cadres such as the nurses, doctors (where available) and traditional birth attendants are providing maternal and newborn health care services in the facilities. Nurses receive limited training on midwifery during their pre-service nursing course and may not receive specific supervision and support on their midwifery work [26, 42]. They are often busy with other work in the facilities such as diagnosis and treatment of illnesses and can spend less time on the midwifery role. This is also the case for doctors. Traditional birth attendants can only take on certain tasks such as assisting trained staff at delivery, health education and supporting women to breastfeed and care for the baby, and need support and supervision from trained staff to carry out these roles. All of these factors have implications for the quality of care that women and babies receive.

It is clear from the analysis of training outputs that it will take a long time to train enough midwives to fill the gaps in facilities across the province. The question to consider is whether to formally shift midwifery tasks to nurses, especially as there are more nurses compared to the authorised posts. This would require additional midwifery training during the pre-service courses and, for those already in post, regular in-service training and high-quality supervision.

### Strengths and limitations

This is the first study to capture the situation of numbers and distribution of SBA cadres in a DRC province. This analysis can help develop appropriate strategies at different levels of the health system to address the HRH challenges in the Province. In many conflict-affected settings, data is often missing and incomplete, so creative ways to gather this data are needed. In this study, the data for 2013 and 2014 was not available in the Provincial Health Division HRH database, so the provincial HRH analyst showed resourcefulness in collecting data directly from the districts.

The lack of data on recruitment and attrition in the provincial health division database is another limitation, as this data could be used to better understand the workforce dynamics. However, if the Provincial Health Division can now see the importance of this data, this will help their workforce planning in the future.

## Conclusion

This is the first study to use existing human resource data to analyse the numbers and distribution of SBA cadres in a DRC province. This has provided insight into the mismatch of supply and demand of midwives, nurses and doctors and has highlighted the extreme shortage of midwives throughout the province. This is the first step, and further investigations are needed to better understand the situation and to be able to develop evidence-informed strategies to ensure a more equitable distribution of SBAs throughout this province and other settings. However, based on this study, we suggest two options for consideration: first, the government should consider providing a rural placement allowance for SBA cadres to improve attraction to and retention in rural areas, and second, provision of additional training and supervision for nurses in rural areas to undertake midwifery tasks. Without ways to get more SBAs in rural areas, Ituri Province and more generally the DRC will continue to struggle to reduce maternal mortality.

## Data Availability

The datasets generated and analysed during the current study are not publicly available as they are kept in the provincial health division office and schools training health professionals. As for the collected data, they are available from the corresponding author on reasonable request.
